# Blood Metabolites and Faecal Microbial Communities in Nonpregnant and Early Gestation Ewes in Highly Cold Areas

**DOI:** 10.3390/biology12111436

**Published:** 2023-11-16

**Authors:** Zhiwu Wu, Yanyan Yang, Biao Wang, Kefyalew Gebeyew, Shaoxun Tang, Xuefeng Han, Zhixiong He, Zhiliang Tan

**Affiliations:** 1 CAS Key Laboratory for Agro-Ecological Processes in Subtropical Region, National Engineering Laboratory for Pollution Control and Waste Utilization in Livestock and Poultry Production, Hunan Provincial Key Laboratory of Animal Nutritional Physiology and Metabolic Process, Institute of Subtropical Agriculture, The Chinese Academy of Sciences, Changsha 410125, China; wuzhiwu21@mails.ucas.ac.cn (Z.W.); kefe@isa.ac.cn (K.G.); sxtang@isa.ac.cn (S.T.); zltan@isa.ac.cn (Z.T.); 2 University of Chinese Academy of Sciences, Beijing 101408, China; 3 Inner Mongolia Academy of Agricultural and Animal Husbandry Sciences, Hohhot 010031, China; swallow_0088@163.com (Y.Y.); wangbnd@163.com (B.W.)

**Keywords:** early gestation, pregnancy, metabolite, faecal microbiota, biomarker

## Abstract

**Simple Summary:**

In this study, we elucidated the changes in metabolites and microbial communities in pregnant ewes during early gestation, and tried to find new biomarkers to predict early pregnancy in sheep. Significant differences were found in the blood biochemical parameters and blood metabolites, but not in the faecal microbial community. At the same time, five potential pregnancy biomarkers were found to predict early pregnancy in ewes. These detected differences will provide a scientific theoretical basis for the rational nutritional regulation of farmers, and these newly discovered biomarkers will promote the development of technology for detecting early pregnancy in sheep in highly cold areas.

**Abstract:**

Ewes undergo complex metabolic changes during pregnancy. Understanding the specific process of these changes is a necessary prerequisite in ewes for regulating and intervening in order to maintain pregnancies. However, there have been relatively few studies on the specific changes that occur in nutritional metabolism in pregnant ewes during early gestation, especially for some landrace ewes in highly cold areas. Therefore, this study aimed to (1) elucidate the changes in metabolites and microbial communities in pregnant ewes during early gestation using metabolomics and 16S ribosomal RNA gene (rDNA) amplicon sequencing approaches, and to (2) discover novel early pregnancy-induced biomarkers in the blood and faeces. Rams were placed together with ewes on D0 and removed on D45. During early gestation, blood and faecal samples were collected from ewes in a highly cold area for analysing the metabolites and microbial communities; these were retrospectively classified as the early gestation pregnant (EP) ewe group or the nonpregnant (NP) ewe group based on the lambing status recorded during the expected delivery period. The differences in the plasma biochemical parameters, plasma metabolites, and faecal microbial communities of pregnant and nonpregnant ewes were characterised. The GC, IL-6, O-acetyl-l-serine, L-glutamine, and 6-acetamido-2-oxohexanoic acid were screened out as potential biomarkers for evaluating the occurrence of early pregnancy. These novel early pregnancy-induced metabolites discovered in ewes might allow for the development of technologies to detect early pregnancies in sheep in highly cold areas.

## 1. Introduction

Breeding ewes is an important basis for flock development, and pregnancy rate is the main factor affecting the reproductive rate of ewes. As sheep are an important source of animal protein, improving the pregnancy rate of ewes will not only improve the economic efficiency of the sector, but will also benefit human diets worldwide [1]. Pregnancy in ewes causes huge physiological and metabolic changes, and is a factor in determining the management of pregnant and nonpregnant ewes [2,3]. Exploring metabolic differences in pregnant and nonpregnant ewes could improve the understanding of the physiological or pathological characteristics and mechanisms in pregnancy. The early identification of pregnancy is important for pregnancy monitoring and animal production.

Ultrasonography stands as the main and most commonly utilized technique for diagnosing pregnancy in ewes [4]. Although this method effectively replaces the traditional method of rectal palpation when judging pregnancies and improves the accuracy of diagnosis, it also has many limitations [5]. Ultrasonography necessitates proficient scanning expertise, and farmers frequently face a deficit in requisite technology and expertise. Consequently, they often engage veterinarians and allied specialists to conduct the procedure, leading to augmented costs and constraints linked to veterinarians’ availability during specific working hours. Thus, there is an urgent need for the identification and application of simpler diagnostic methods for pregnancy; among these, molecular markers have been widely studied by researchers.

In recent studies, some molecular markers have been determined to have the potential to diagnose pregnancy at early gestation, including pregnancy-associated glycoproteins [6], blood and urine metabolites [1], faecal microbiota [7], specific genes [8], and circulating microRNAs [9]. However, biomarkers for early pregnancy diagnosis in sheep can be affected by factors such as breed, detection time, and even temperature. Studies have shown that cold stress may lead to animal reproduction impairment and reproductive hormone disturbance [10,11], which may mean that there might be some differences in metabolism between animals in highly cold areas and those in non-cold areas. At present, the identification of biomarkers for the diagnosis of early pregnancy in sheep in highly cold areas has not been reported. In addition, most studies that have identified biomarkers of early pregnancy in sheep have focused on hormones or individual metabolites associated with pregnancy [12], with little focus on nonhormonal metabolic changes. Moreover, no attempts have been made to identify biomarkers of pregnancy among sheep faecal microbes.

In the present study, we hypothesised that blood and faecal biomarkers could serve as potential markers to predict sheep pregnancy at early gestation. Therefore, we aimed to (1) elucidate the changes in metabolites and microbial communities in pregnant ewes during early gestation using plasma metabolomics and 16S ribosomal RNA gene (rDNA) amplicon sequencing approaches, and to (2) discover novel early pregnancy-induced biomarkers in blood and faeces. The metabolites and microorganisms differentially expressed between pregnant and nonpregnant ewes during early gestation may serve as the basis for identifying novel pregnancy biomarkers for ewes in highly cold areas.

## 2. Materials and Methods

### 2.1. Animals

**Figure 1:**
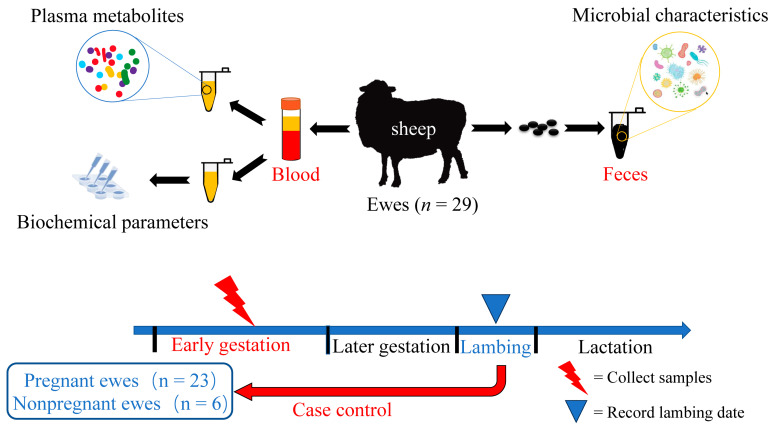
A total of 29 healthy ewes with similar body weights were selected and fed according to the local feeding nutrition standards and methods for meat sheep. On D90, blood and faeces were collected for metabolites and microbial communities from ewes in highly cold areas, and the ewes were retrospectively classified as the early gestation pregnant (EP) ewe group or nonpregnant (NP) ewe group according to whether the ewes were lambing before the latest lambing period. The ewes in the EP group had a similar delivery date, while the ewes in the NP group were those that were not pregnant in the sheep herd. Blood samples were used for phenotypic parameters and plasma untargeted metabolome analysis, and faecal samples were used for 16S rDNA amplicon sequencing and analysis.

All of the procedures performed in this study were in accordance with the guidelines for animal care and use, and were approved (no. ISA000263) by the Animal Ethics Committee of the Institute of Subtropical Agriculture, Chinese Academy of Sciences, Changsha, China. The experimental animals were female Hulunbuir sheep, a meat sheep breed, in the Inner Mongolia Autonomous Region, China. The animals were raised at the Ewenke Experiment Station of the Chinese Academy of Sciences, Ewenke County (118°48′02″–121°09′25″ E, 47°32′50″–49°15′37″ N), China. This experiment was conducted from September 2021 to January 2022, and the average temperature was −7.51 °C. The animals were group-housed, provided with ample water, and fed twice a day (08:00 a.m. and 17:00 p.m.) with an average feeding amount of 2 kg/d per ewe, consisting of 1.5 kg/d alfalfa and 0.5 kg/d concentrate. The ingredients and chemical composition of the diet are shown in Supplementary Table S1. During the mating period, the rams were introduced into the ewe population for 45 days for free mating (on D0). After the rams were removed (on D45), blood and faecal samples were collected from the ewes on D90 (at early gestation; Figure 1). During the expected delivery period, the pregnant ewes delivered and their lambing dates were accurately recorded. Based on the lambing status recorded during the lambing stage, a total of 29 ewes (24 months old) were retrospectively classified into the early gestation pregnant (EP, *n* = 23) ewe group or the nonpregnant (NP, *n* = 6) ewe group. The ewes in the EP group had similar delivery dates and the ewes in the NP group were those in the sheep herd that were not pregnant.

### 2.2. Sample Collection

#### 2.2.1. Blood Sample Collection

Before blood collection, all of the animals were tested by veterinarians to ensure that they were free of disease. Fasting blood collection was carried out before the morning feeding on the last experimental day. Tubes containing anticoagulant heparin sodium and a venous blood collection needle were used to collect jugular blood samples from the ewes. The blood collection vessels were gently inverted several times to fully mix the anticoagulant and blood. The plasma was promptly separated by centrifuging the blood samples at 3000× *g* for 15 min. Then, the plasma was aliquoted into sterile Eppendorf 1.5-mL tubes and preserved at −80 °C.

#### 2.2.2. Faecal Sample Collection

Faecal samples were collected for microbial analysis by an experimenter’s finger wearing a sterilised glove, which was inserted approximately 5 cm into the anus. The ewes’ tails were lifted manually by a gloved sampling technician to remove the faecal samples from the anus carefully, ensuring no contact with any surfaces so as to avoid contamination. The samples were then deposited into sterile 15-mL centrifuge tubes. (RNase-free, DNase-free Speciality). The centrifuge tubes were hermetically sealed and promptly chilled on ice for transportation to a −80 °C refrigerator in the laboratory until DNA extraction was performed.

### 2.3. Measurement of Blood Biochemical Parameters, Mineral and Hormonal Concentrations, and Immune Parameters

For the blood samples of 23 ewes in the EP group, the obtained plasma of 3 ewes was insufficient to measure the blood biochemical parameters, mineral and hormonal concentrations, and immune indices. Therefore, for the EP group, the plasma samples of only 20 ewes were measured for these blood indicators. Before testing, the blood samples were thawed at 4 °C on ice. The plasma total protein (TP), albumin (ALB), alanine transaminase (ALT), aspartate transaminase (AST), alkaline phosphatase (ALP), gamma glutamyl transpeptidase (GGT), cholinesterase (CHE), direct bilirubin (DBIL), total bilirubin (TBIL), triglycerides (TG), total cholesterol (CHOL), low-density lipoprotein cholesterol (LDL-C), high-density lipoprotein cholesterol (HDL-C), glucose (GLU), amylase (AMS), D-lactic acid (LAC), lactate dehydrogenase (LDH), blood urea nitrogen (BUN), and creatinine (CRE) were measured on a Mindray BS-230 automatic chemistry analyser (Shenzhen, China). The plasma concentrations of free fatty acids (FFAs) and β-hydroxybutyrate (BHB) were determined using commercial spectrophotometric diagnostic kits (Solarbio, Beijing, China) according to the manufacturer’s instructions. The plasma concentrations of magnesium (Mg), ferrum (Fe), calcium (Ca), and phosphorus (P) were determined using a Mindray BS-230 automatic chemistry analyser (Shenzhen, China).

To evaluate the endocrine status, the concentrations of estradiol (E_2_), progesterone (P_4_), FSH, and LH in the plasma were also detected with the Multimode Microplate Reader (Infinite M200 PRO Multimode, Tecan, Swiss) and a commercial enzyme-linked immunosorbent assay (ELISA) kit (CUSABIO, Wuhan, China), according to the manufacturers’ protocols.

For the immune parameters, the plasma concentrations of interleukin 1β (IL-1β), interleukin 6 (IL-6), tumour necrosis factor (TNF-α), immunoglobulin G (IgG), immunoglobulin A (IgA), and immunoglobulin M (IgM) were determined with the Multimode Microplate Reader (Infinite M200 PRO Multimode, Tecan, Männedorf, Switzerland) and a commercial ELISA kit (CUSABIO, Wuhan, China), according to the manufacturer’s protocols.

### 2.4. Metabolomics Profiling of Sheep Plasma

#### 2.4.1. Plasma Metabolite Extraction

Low-molecular-weight metabolites (<1000 Da) were separated from the plasma samples by methanol precipitation. Each frozen plasma sample was thawed in the refrigerator at 4 °C. A volume of 100 μL from each sample was then transferred to a 1.5 mL sterile Eppendorf tube. Next, 700 µL of the extracting solution containing the internal standard 1 (methanol:acetonitrile:water = 4:2:1, *v*/*v*/*v*) was added to the tube. The mixture was shaken for 1 min and placed in a −20 °C freezer for 2 h. Following this, the tube was centrifuged at 4 °C at 25,000× *g* for 15 min, and 600 μL of the supernatant was transferred into new 1.5 mL sterile Eppendorf tubes. Then, 180 µL of the compound solvent (methanol:pure water = 1:1, *v*/*v*) was added and swirled for 10 min until fully mixed and dissolved for measurement. Equal amounts (20 μL) from each plasma sample were combined to create quality control (QC) samples before the analysis preparation. The pooled sample was utilized to establish a representative average profile encompassing all of the encountered analytes in the analysis.

#### 2.4.2. Ultra-Performance Liquid Chromatography (UPLC)–Mass Spectrometry (MS) Experiments

In this experiment, a Waters UPLC I-Class Plus (Waters, Milford, MA, USA) Tandem Q Exactive High-Resolution Mass Spectrometer (Thermo Fisher Scientific, Waltham, MA, USA) was employed to separate and detect the metabolites.

Chromatographic separation occurred on a Waters ACQUITY UPLC BEH C18 column (1.7 μm, 2.1 mm × 100 mm, Waters, USA) maintained at 45 °C. In the positive mode, the mobile phase comprised 0.1% formic acid (A) and acetonitrile (B), while in the negative mode, it consisted of 10 mM ammonium formate (A) and acetonitrile (B). The gradient conditions were as follows: 0–1 min, 2% B; 1–9 min, 2–98% B; 9–12 min, 98% B; 12–12.1 min, 98–2% B; and 12.1–15 min, 2% B. The flow rate was set at 0.35 mL/min, and 5 μL was injected for analysis.

Q Exactive was employed for both the primary and secondary mass spectrometry data acquisition. The analysis encompassed a full scan range of 70–1050 *m*/*z* with a resolution of 70,000. The automatic gain control (AGC) target for MS acquisitions was established at 3 × 10^6^, and the maximum ion injection time was set to 100 ms. The top three precursors were chosen for subsequent tandem mass spectrometry (MS–MS) fragmentation, employing a maximum ion injection time of 50 ms and achieving a resolution of 17,500, with an AGC set at 1 × 10^5^. Stepped normalised collision energies of 20, 40, and 60 eV were applied. Among these operational parameters, the sheath gas flow rate was established at 40 arb, with the auxiliary gas flow rate set at 10 arb. In the positive ion mode, the spray voltage was maintained at 3.80 kV, while in negative ion mode, the spray voltage was adjusted to 3.20 kV. The capillary temperature was carefully maintained at 320 °C, and the auxiliary gas heater temperature was consistently maintained at 350 °C.

#### 2.4.3. Metabolome Data Analysis

After importing the MS data offline into Compound Discoverer 3.3 software (Thermo Fisher Scientific, USA), an analysis was conducted by cross-referencing the MS data with the bmdb (BGI metabolome), mzcloud, and chemspider online databases. This approach yielded a data matrix encompassing crucial information such as metabolite peak areas and identification results. The resultant UPLC–MS data were imported into the R language analysis package for orthogonal partial least squares-discriminant analysis (OPLS-DA). Additionally, the imported data underwent t-test and fold change analysis utilizing the R language analysis package for univariate analysis. The gathered data were further processed employing OPLS-DA, a supervised analysis method renowned for providing a comprehensive reflection of differences among the experimental samples. OPLS-DA was applied to construct a relational model correlating higher abundance levels with experimental samples, facilitating the development of predictive models for distinct samples. The outcomes were visually represented through score plots, effectively showcasing group clustering patterns. Concurrently, variable importance of projection (VIP) values was calculated as part of the OPLS-DA model. The VIP value computation served to gauge the influence intensity and explanatory capacity of each metabolite’s higher abundance mode on sample group differentiation. The robustness of multivariate statistical analysis (MVA) models underwent evaluation through cross-validation analysis of variance (CV-ANOVA) and assessment of the R^2^Y and Q^2^ values. The VIP value was employed as a screening criterion to identify potential biomarkers associated with the sexual maturation process. Furthermore, in the univariate analysis, fold change (FC) analysis was conducted. The FC and corresponding *p*-values were visualized in either txt format or through a volcano plot. Potential metabolic biomarkers were identified based on criteria including a VIP > 1, *p* < 0.05, and FC > 1.2 / FC < 0.83. Hierarchical clustering was carried out utilizing the Heatmap Illustrator tool, and a Wayne figure was generated employing the Venny tool (version 2.1). For metabolic pathway analysis, the Kyoto Encyclopaedia of Genes and Genomes (KEGG, www.kegg.jp/kegg/pathway.html, accessed on 20 July 2021) was employed. The metabolites’ discriminative capacity was evaluated through the area under the receiver operating characteristic (ROC) curve (AUC).

### 2.5. 16S rDNA Amplicon Sequencing and Analysis

Bacterial DNA was extracted from faecal samples utilizing the QIAamp Fast DNA Stool Mini Kit (Qiagen, Hilden, Germany), following the manufacturer’s instructions. The DNA concentration was determined using a fluorometer, and the sample integrity was assessed through agarose gel electrophoresis. For constructing the PCR-based library for 16S rDNA amplicon sequencing, the V3–V4 dual-index fusion PCR primer cocktail and PCR master mix (NEB Phusion high-fidelity PCR master mix) were utilized in the PCR amplification process. The PCR amplification products underwent purification utilizing Agencourt Ampure XP beads to eliminate non-specific products. Subsequently, the qualified libraries were subjected to paired-end sequencing employing the PE300 sequencing approach on the Illumina HiSeq 2500 system. Before conducting the 16S rDNA data analysis, raw sequences underwent filtration using an in-house program. Paired-end reads were then combined into the tag using FLASH (Fast Length Adjustment of Short reads, v1.2.11, http://www.cbcb.umd.edu/software/flash) (accessed on 17 August 2023). On average, 30,877 ± 791 tags were analysed using scripts within the USEARCH software (v7.0.1090, https://www.drive5.com/usearch/) (accessed on 17 August 2023). Specifically, the operational taxonomic units (OTUs) were formed by clustering these tags with a 97% threshold utilizing UPARSE [13], leading to the acquisition of distinct representative sequences for each OTU. The removal of chimeras was conducted through UCHIME v4.2.40 (http://drive5.com/uchime) (accessed on 17 August 2023). Taxonomic classification of OTU representative sequences was achieved using the Ribosomal Database Project (RDP) Classifier v.2.2 trained on the Greengenes database v201305, implemented via QIIME2 (https://qiime2.org/) (accessed on 17 August 2023). The OTU table was utilized for the computation of beta diversities and the provision of taxonomic profiles. Subsequent statistical analysis was executed within R software version 3.0.1 (R Core Team, Vienna, Austria).

Using Mothur software version 1.31.2, (https://www.mothur.org) (accessed on 17 August 2023), richness and diversity analyses were conducted with the four indicators of Chao1, Ace, Shannon, and Simpson. Beta diversity was tested in QIIME v.1.80 (http://qiime.sourceforge.net/) (accessed on 17 August 2023) [14] to compare the differences between different samples. Principal coordinates analysis (PCoA) was used to visualise the results. A two-tailed Wilcoxon rank-sum test was utilized to examine the differential abundances of phyla and genera among the EP and NP individuals.

### 2.6. Statistical Analyses

Shapiro–Wilk and Levene’s tests were conducted to validate the normality and homoscedasticity of the data, respectively. The plasma biochemical and immune parameters, mineral and hormonal concentrations, metabolite peak-intensity results, and alpha-diversity indexes were analysed using the independent-sample *t*-test in SPSS software (SPSS version 27.0, SPSS, Inc., Chicago, IL, USA). The assessment of the relative abundances of bacteria at both phylum and genus levels was carried out through the Wilcoxon rank-sum test. Data were presented as means ± SEM. Statistical significance was attributed to *p* < 0.05, while a range of 0.05 ≤ *p* < 0.10 was considered as a statistical tendency.

## 3. Results

### 3.1. Plasma Biochemical Parameters, Mineral and Hormonal Concentrations, and Immune Parameters

Regarding biochemical parameters, the EP group had lower plasma levels of AST (*p* = 0.027), LDH (*p* = 0.007), TG (*p* = 0.031), and CHE (*p* < 0.001) compared with the NP group (Table 1). Regarding the mineral element concentrations, compared with the NP group, the EP group had lower levels of plasma Mg (*p* = 0.003) and Fe (*p* = 0.032) (Table 2). Regarding the hormonal concentrations, compared with the NP group, the EP group had higher levels of E_2_ (*p* = 0.012) and glucocorticoid (GC, *p* = 0.002), and trended towards a higher level of P_4_ (*p* = 0.097; Table 3). Regarding the immune parameters, the IL-6 level in the EP group was significantly higher than that in the NP group (*p* < 0.001; Table 4).

### 3.2. Metabolomic Profiling

**Figure 2:**
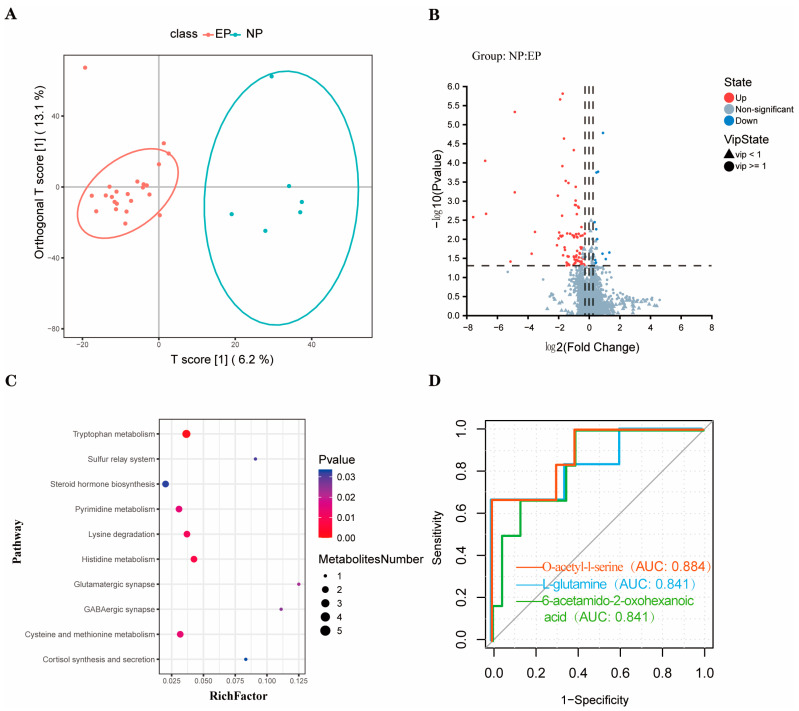
Nontargeted metabolomics profile of plasma samples of sheep in the EP and NP groups. (**A**) OPLS-DA analysis of differential metabolites (The red and blue curves represent the confidence intervals for each group). (**B**) Volcano diagram of differential metabolites. (**C**) Differential metabolic pathways. (**D**) ROC curve (The red line means O-acetyl-l-serine, the blue line means L-glutamine, the green line means 6-acetamido-2-oxohexanoic acid). NP = nonpregnant ewe group; EP = early gestation pregnant ewe group.

A total of 2015 metabolites were detected in the plasma of ewes using untargeted UPLC–MS analysis, comprising 1662 in the electrospray ionisation positive mode and 353 in the negative mode. In this study, the supervised OPLS-DA multivariable method was used to visualise the stratification of samples, which revealed that there was a significant separation between the two groups (Figure 2A). The quality of the OPLS-DA analysis model was evaluated by CV-ANOVA, which showed good fitting (R^2^Y = 0.870, Q^2^ = 0.153). With the values of *p* < 0.05, VIP > 1, FC < 0.83, and FC < 1.20, 99 metabolites were found to be significantly different between the two groups, including 82 metabolites enriched and 17 metabolites depleted in the EP group (Figure 2B). The significantly enriched pathways for the 99 differential metabolites between the two groups included tryptophan metabolism, histidine metabolism, lysine degradation, cysteine and methionine metabolism, and pyrimidine metabolism (Figure 2C).

**Figure 3:**
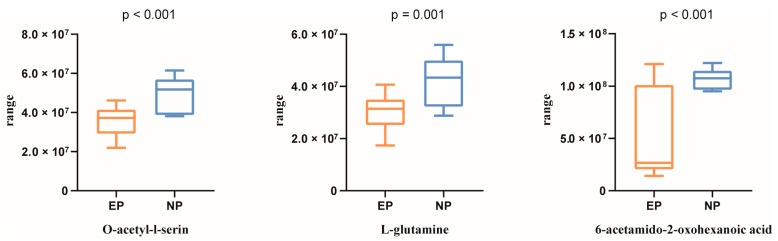
Peak-intensity diagram of three metabolites. NP = nonpregnant ewe group; EP = early gestation pregnant ewe group. *p* < 0.05 is regarded as statistically significant.

Based on the above five differentially regulated pathways, the metabolites with AUC > 0.80 were selected. Three typical metabolites were identified as follows (Figure 2D): O-acetyl-l-serine (AUC = 0.884), L-glutamine (AUC = 0.841), and 6-acetamido-2-oxohexanoic acid (AUC = 0.841). O-acetyl-l-serin, L-glutamine, and 6-acetamido-2-oxohexanoic acid were then studied using UPLC-MS peak-intensity diagram analysis, and it was found that the three metabolites were significantly differentially produced between the EP and NP ewes (Figure 3).

### 3.3. Faecal Microbial Diversity

**Figure 4:**
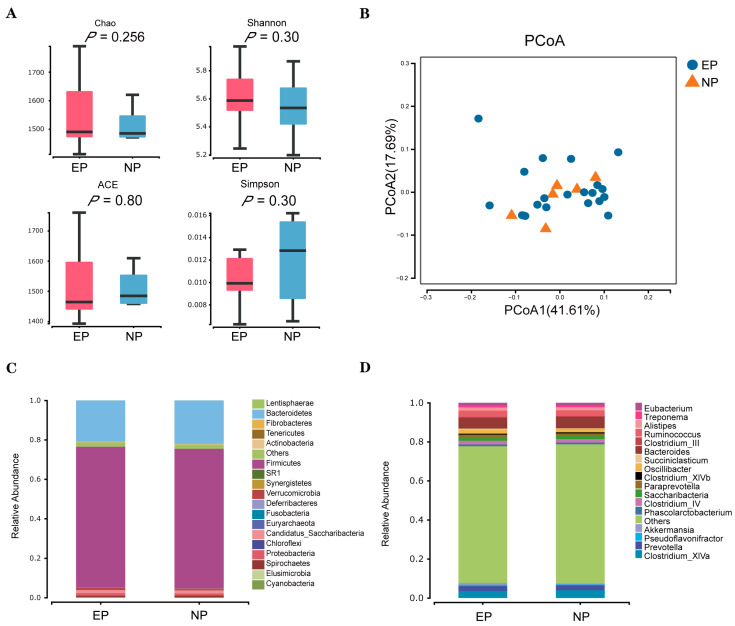
Microbial communities in faecal samples of sheep in the EP and NP groups. (**A**) Alpha diversity waves over time. Four alpha diversity (ACE, Chao, Simpson, and Shannon) metric indices are depicted as boxplots coloured by timepoint. (**B**) Weighted PCoA plot of β diversity. (**C**) Relative abundance at the phylum level. (**D**) Relative abundance at the genus level. NP = nonpregnant ewe group; EP = early gestation pregnant ewe group.

Using the 16S amplicon sequencing platform, we detected and clustered microorganisms from the faecal samples. A total of 2,058,246 reads were obtained from 27 samples; after quality control, 2,032,996 clean reads were retained. The sequences were grouped as OTUs with similarities above 97%. The numbers of OTUs in each sample ranged from 1001 to 1591. PCoA using the weighted UniFrac distance showed that no distinct separations (*p* > 0.05; Figure 4A) were formed between the EP and NP groups. Moreover, alpha diversity indices revealed no significant differences between the two experimental groups (*p* > 0.05; Figure 4B). A total of 19 phyla were detected from 27 faecal samples from the ewes (Figure 4C), and the phyla Firmicutes and Bacteroidetes were the dominant flora in both groups. Furthermore, 164 genera were identified at the genus level from the 27 faecal samples of ewes, among them, Bacteroides showed the highest relative abundance in the faecal samples, followed by *Clostridium_XlVa*, *Ruminococcus*, *Prevotella*, and *Oscillibacter* (Figure 4D).

## 4. Discussion

Blood biochemical parameters can usually be used for disease diagnosis and evaluations of animal health status [15]. Studies have shown that pregnancy can cause changes in a variety of blood biochemical parameters [16]. The results of our study might provide strong support for this view.

AST is an enzyme abundantly present in the liver and heart muscles, which plays a crucial role in amino acid metabolism [17]. In the present study, the plasma AST concentrations in the EP group were lower than those in the NP group, indicating differences in amino acid metabolism in pregnant ewes. Lactate dehydrogenase, a glycolytic enzyme, facilitates the conversion of lactate into pyruvate—an essential step in cellular energy production. The LDH value was lower in the EP group than in the NP group, suggesting a decrease in pyruvate in the early pregnant ewes, which further indicated that the processes of glucose metabolism and energy production in the EP ewes were inhibited. TGs mainly participate in the processes of lipid decomposition and metabolism in the body, providing energy. The plasma TG content of the EP group was significantly lower than that of the NP group, indicating that the body lipid metabolism of pregnant ewes changed dramatically during early gestation. Cholinesterase is a type of glycoprotein synthesised in the liver and is regarded as an important physiological parameter reflecting liver function. It has been suggested that the physiological role of CHE in the blood may involve delaying choline metabolism, especially during embryonic development [18]. The decrease in the plasma CHE in the EP group compared with the NP group indicated decreasing choline metabolism.

Changes in mineral element concentrations in the blood reflect the nutritional status of animals very well. Among them, Mg is an essential constant element in bones and soft tissues, while P and Ca play important roles in kidney metabolism and skeletal growth and development [19]. Many recent reports and studies have confirmed the phenomenon of Mg deficiency during gestation [20,21]. The results of this study were consistent with those of previous studies, with significantly lower plasma Mg levels in the EP group than in the NP group, confirming that pregnancy may increase the body’s requirement for Mg. Fe is a necessary trace element for the normal operation of the animal body and is equally important for the occurrence of pregnancy [22]. In the early stages of pregnancy, increases in foetal development and haematopoiesis lead to a large amount of Fe consumption [23]. This phenomenon is consistent with the results from this study, that the plasma Fe content of the pregnant ewes was lower than that of the nonpregnant ewes.

Hormones are the main chemicals that regulate the growth, development, and metabolism of animals. E_2_ can promote the differentiation of uterine glands in the body, ensuring the occurrence of normal pregnancy [24]. In our study, the results showed that the E_2_ levels in pregnant ewes were higher than those in nonpregnant ewes during early gestation, similar to previous studies [25]. The cause for this difference might be that large amounts of E_2_ and P_4_ are produced in pregnant female animals to maintain a normal pregnancy [26]. In animals, GC, as a major hormone from alpha cells, coordinates with INS to maintain blood glucose balance. Several human and rat studies have reported elevated levels of glucagon in the maternal blood at some point during pregnancy [27,28]. Furthermore, another study claimed that pregnancy increases the proliferation and mass of alpha-cells in the pancreas, resulting in higher levels of glucagon [29]. In the experimental results, the GC content in the EP group was higher than that in the NP group, which may indicate the hypoglycaemic state of ewes in early pregnancy and the potential for GC as a diagnostic marker in the early pregnancy of ewes.

Cytokines are active substances that regulate immune function in the body and are produced by activated lymphocytes. In this experiment, we detected differences in IL-6 between the EP and NP groups, indicating partial differences in maternal immune levels during early gestation. In the animal body, IL-6 possesses the ability to modulate the growth and differentiation of diverse cell types, as well as regulate immune responses, acute phase reactions, and hematopoietic function, thereby contributing significantly to the body’s anti-infective immune response [30]. According to previous studies, some innate and pro-inflammatory factors of the body’s immunity during pregnancy will be induced, which includes a significant promoting effect on the production of IL-6 [31,32]. A recent study also demonstrated the central role of the immune system in mediating a successful pregnancy, and the upregulation of inflammatory factors such as IL-6 caused by implantation is highly conserved in many mammals [33]. Thus, IL-6 may be an overlooked marker with potential to help determine pregnancy, although the specific mechanisms and influencing factors of changes in the immune levels in ewes in early pregnancy need to be further explored.

In terms of metabolites, the EP group could be clearly distinguished from the NP group by untargeted metabolomic analysis, indicating that pregnancy did cause some distinct metabolic differences in the ewes’ body during early gestation. Ninety-nine metabolites showed key differences between the EP and NP groups in our discovery cohort. Pathway analysis revealed that these metabolites were mainly enriched in five metabolic pathways, namely, tryptophan metabolism, histidine metabolism, lysine degradation, cysteine and methionine metabolism, and pyrimidine metabolism. Similar to the results of this study, in a study evaluating Holstein cows at the beginning of pregnancy, pregnancy-related plasma metabolites were also affected by changes in tryptophan metabolism [34]. Some products of tryptophan metabolism, such as o-aminobenzoic acid and 5-hydroxytryptophan, are closely related to the successful completion of mammalian pregnancy [35,36]. Lysine degradation, histidine synthesis, glutamine synthesis, and other processes are downregulated in pregnant ewes, suggesting their metabolic adaptation during early pregnancy, which is consistent with the findings shown in pregnant humans [37,38]. In short, the metabolomics results mainly revealed that the metabolite changes in pregnant ewes during early pregnancy were first reflected in amino acid metabolism. Subsequent studies can further determine the specific amino acid content, which will be a possible direction for the development of a pregnancy field test.

Regarding microorganisms, in the present study, the results showed no significant differences in the diversity of microbiota in faecal samples from the EP and NP groups during early pregnancy, which is consistent with the findings of some studies [39,40]. However, other studies reported results quite different from ours [41]. We speculate that these differences in findings may be related to differences in ewes’ age and parity [42]. In addition, some other studies have shown that the animal microbiota can be influenced by genetic factors, pregnancy, temperature, climate, and other environmental factors [43]. The experimental animals used in this study were in a highly cold area during the experimental period; thus, cold conditions may be another factor influencing the results. Some studies have shown that cold stress has significant impacts on animal production, including physiological, reproductive, and health aspects [44]. According to previous research, some bacterial genera, such as *Fibrobacter*, *Ruminococcus*, *Anaerobes*, and *Lactobacillus*, are good at overcoming adverse climatic conditions and exhibit a higher abundance in cold spring and winter seasons [45]. Therefore, given the high complexity of intestinal microbiota changes and their susceptibility to various internal and external factors, the results of this study also suggest the difficulty in developing microbial markers for the diagnosis of early pregnancy in pregnant ewes, especially in highly cold areas.

In previous studies, efforts have been made to identify pregnancy in ewes at an early stage. For instance, alterations in the expression levels of interferon-tau-stimulated genes within both the thymus [46] and endometrium [47] were recognized as indicative of a pregnancy signal during early gestation. Other studies have shown that some signature proteins identified in the uterine fluid can also be used as viable indicators of pregnancy [48]. However, none of these gene and protein markers have been validated by rigorous ROC curve analysis, nor have they been tested in actual production. Pregnancy-associated glycoprotein serves as a reliable protein biomarker to assess pregnancy in ewes, detectable as early as 30 days into gestation [49]; however, it is limited in species applicability, and its sensitivity may be skewed by pregnancy rate [50]. In brief, some previous studies still have certain limitations and shortcomings, and the discovery of new biomarkers in this study will provide a supplement for the diagnosis of early pregnancy in sheep.

O-acetyl-l-serine, a precursor essential for cysteine biosynthesis, serves as a pivotal intermediate in the metabolic pathways of cysteine and methionine. Cysteine and methionine, indispensable amino acids in the body, are subject to influence from various factors such as physiological stage and methylation donors or acceptors, among others [45]. Previous work showed that the plasma concentrations of total cysteine and homocysteine were lower during gestation [51]. However, cysteine and methionine are affected by many factors in nutrient metabolism, and the complex mechanisms regulating these differences are still unclear [52]. The results of this study showed that O-acetyl-l-serine was downregulated in the EP group, indicating that the cysteine synthesis process in the plasma of pregnant ewes was inhibited during early gestation, which could be related to the body’s metabolic level and changes in diet.

L-glutamine, the amino acid with the highest abundance and broadest utilization within the body, holds significant importance in intermediary metabolism, immune function, and maintaining pH homeostasis [53]. Glutamine can also act as a substrate for different biosynthetic pathways to maintain cellular integrity and function in almost all cells [54]. During gestation, an ewe’s body undergoes complex metabolic changes, and the metabolic rates of cells accelerate in various tissues. As expected, the results of the present study showed that L-glutamine was downregulated in ewes during early pregnancy, which may indicate increased consumption of L-glutamine to support foetal growth and development, maintain immune function, and promote tissue repair.

The product 6-acetamido-2-oxohexanoic acid is important in the lysine degradation process. Lysine is an essential amino acid that plays important roles in mammalian growth and development [55]. In cells, lysine can produce 6-acetamido-2-oxohexanoic acid through its degradation pathway, which plays a critical role in cellular metabolism. Lysine not only participates in protein synthesis and repair within organisms, but also acts as a fundamental component in various metabolic pathways and physiological functions. In this study, 6-acetamido-2-oxohexanoic acid was downregulated in the EP group, indicating that the decomposition and utilisation of lysine were inhibited and suggesting that protein synthesis might be influenced during gestation.

## 5. Conclusions

We found differences in plasma biochemical parameters, mineral and hormonal concentrations, immune parameters, and metabolic levels between the pregnant and nonpregnant ewes during the early gestation period, with no significant differences in microbial level. Moreover, two biomacromolecules (GC and IL-6) and three small molecule metabolites (O-acetyl-l-serine, L-glutamine, and 6-acetamido-2-oxohexanoic acid) were screened out as potential biomarkers for evaluating the occurrence of early pregnancy. The discovery of these new early pregnancy biomarkers in the plasma could be used to develop new methods for predicting positive pregnancy in sheep that are simpler and more convenient than traditional diagnostic methods. The current findings would prompt the formulation of a hypothesis regarding new molecules and mechanisms for early pregnancy signalling and recognition in sheep in highly cold areas.

## Figures and Tables

**Table 1:** Differences in plasma biochemical parameters in early gestation pregnant and nonpregnant ewes.

Parameters	NP (*n* = 23)	EP (*n* = 6)	SEM	*p*
TP (g/L)	83.9	83.4	3.13	0.891
ALB (g/L)	38.2	37.1	1.36	0.437
BUN (mmol/L)	7.62	6.81	0.60	0.192
ALT (U/L)	16.4	11.9	2.77	0.115
AST (U/L)	120	98.5	9.13	0.027
ALP (U/L)	114	130	27.9	0.570
GGT (U/L)	57.0	50.7	5.25	0.242
CHE (mmol/L)	240	172	13.2	<0.001
DBIL (U/L)	0.80	0.85	0.23	0.838
TBIL (mmol/L)	1.88	2.62	0.51	0.160
TG (mmol/L)	0.27	0.18	0.04	0.031
CHOL (mmol/L)	1.75	1.71	0.11	0.711
LDL-C (μmol/L)	0.67	0.71	0.08	0.617
HDL-C (mmol/L)	1.04	0.99	0.05	0.415
FFA (μmol/L)	150	184	42.9	0.432
BHB (mmol/L)	0.75	0.72	0.08	0.735
GLU (mmol/L)	4.25	4.06	0.44	0.665
AMS (mmol/L)	51.0	24.5	35.0	0.482
LAC (μmol/L)	9.12	9.63	1.74	0.769
LDH (U/L)	542	449	31.7	0.007
CRE (μmol/L)	79.8	87.2	6.71	0.283

TP = total protein; ALB = albumin; ALT = alanine transaminase; AST = aspartate transaminase; ALP = alkaline phosphatase; GGT = gamma glutamyl transpeptidase; CHE = cholinesterase; DBIL = direct bilirubin; TBIL = total bilirubin; TG = triglyceride; CHOL = total cholesterol; LDL-C = low-density lipoprotein cholesterol; HDL-C = high-density lipoprotein cholesterol; FFA = free fatty acid; BHB = β-hydroxybutyrate; GLU = glucose; AMS = amylase; LAC = D-lactic acid; LDH = lactate dehydrogenase; BUN = blood urea nitrogen; CRE = creatinine. NP = nonpregnant ewe group; EP = early gestation pregnant ewe group; SEM = standard error of the mean.

**Table 2:** Differences in plasma mineral element concentrations in early gestation pregnant and nonpregnant ewes.

Parameters	NP (*n* = 23)	EP (*n* = 6)	SEM	*p*
Mg (μmol/L)	1.11	0.90	0.06	0.003
Fe (mmol/L)	29.3	24.6	2.06	0.032
Ca (mmol/L)	2.50	2.37	0.10	0.220
P (U/L)	1.61	1.82	0.20	0.291

Mg = magnesium; Fe = ferrum; Ca = calcium; P = phosphorus. NP = nonpregnant ewe group; EP = early gestation pregnant ewe group; SEM = standard error of the mean.

**Table 3:** Differences in plasma hormonal concentrations in early gestation pregnant and nonpregnant ewes.

Parameters	NP (*n* = 23)	EP (*n* = 6)	SEM	*p*
INS (μIU/mL)	3.43	2.99	0.35	0.263
GC (pg/mL)	1076	1575	139	0.002
LH (mIU/mL)	4.60	1.15	2.79	0.268
FSH (mIU/mL)	0.26	0.25	0.21	0.940
P_4_ (ng/mL)	2.19	4.01	1.30	0.097
E_2_ (pg/mL)	204	527	115	0.012

INS = insulin; GC = glucagon; LH = luteinizing hormone; FSH = follicle-stimulating hormone; P_4_ = progesterone; E_2_ = estradiol. NP = nonpregnant ewe group; EP = early gestation pregnant ewe group; SEM = standard error of the mean.

**Table 4:** Differences in plasma immunity parameters in early gestation pregnant and nonpregnant ewes.

Parameters	NP (*n* = 23)	EP (*n* = 6)	SEM	*p*
IL-1β (pg/mL)	83.2	122	83.0	0.643
IL-6 (pg/mL)	21.2	64.6	10.2	<0.001
TNF-α (pg/mL)	8.15	7.88	0.29	0.348
IgG (g/L)	11.3	13.3	2.90	0.511
IgA (g/L)	1.20	0.92	0.21	0.195
IgM (g/L)	1.35	1.45	0.27	0.719

IL-1β = interleukin 1β; IL-6 = interleukin 6; TNF-α = tumour necrosis factor; IgG = immunoglobulin G; IgA = immunoglobulin A; IgM = immunoglobulin M. NP means the nonpregnant ewe group; EP = early gestation pregnant ewe group; SEM = standard error of the mean.

## Data Availability

The metadata of blood metabolism used in the present study are freely accessible at https://doi.org/10.57760/sciencedb.10327 in the Science Data Bank, and the sequencing data have been uploaded to NCBI, which can be accessed with NO.PRJNA1006002 (accessed on 17 August 2023).

## Supplementary Materials

The following supporting information can be downloaded at: https://www.mdpi.com/article/10.3390/biology12111436/s1, Table S1: Ingredient and chemical composition of diet fed during the experiment (DM basis).
