# Supplementary material for: Blood Metabolites and Faecal Microbial Communities in Nonpregnant and Early Gestation Ewes in Highly Cold Areas
Source: Biology (Basel). 2023 Nov 16;12(11):1436. doi: 10.3390/biology12111436 (PMC10669436; doi:10.3390/biology12111436)
Supplement: Supplementary file 1 [file biology-12-01436-s001.zip › biology-2663744 supplementary.pdf]

Blood Metabolites and Faecal Microbial Communities in Nonpregnant and Early Gestation Ewes in Highly Cold Areas

Zhiwu Wu [1,2], Yanyan Yang [3], Biao Wang [3], Kefyalew Gebeyew [1,2], Shaoxun Tang [1], Xuefeng Han [1,2*], Zhixiong He [1,2*], Zhiliang Tan [1,2]

Table S1.

Ingredient and chemical composition of diet fed during the experiment (DM basis)

	Formula concentrate
**Ingredients, %**	
Corn	57.86
Barley	13.46
Wheat bran	9.20
Soybean meal	3.56
Cottonseed meal	8.13
Premix	5.25
Beet molasses	2.54
Total	100
Nutritional level	
DM (%)	93.06
ASH (%)	8.03
EE (%)	9.78
NDF (%)	14.36
ADF (%)	4.96
CP (%)	17.78
Ca (%)	1.48
P (%)	0.68
GE (MJ/Kg)	15.98

The premix is provided with: Iron 29.45 mg, copper 7.36 mg, zinc 38.65 mg, manganese 26.69 mg, iodine 0.74 mg, cobalt 0.15 mg, selenium 0.06 mg, vitamin A 2310 IU, vitamin D 280 IU, vitamin E 23 IU per kg feed.
